# Surgical treatment of recurrent postoperative discal pseudocyst: A case report and literature review

**DOI:** 10.1097/MD.0000000000031756

**Published:** 2022-11-11

**Authors:** Hong Wang, Shuang Wang, Hailong Yu, Yu Chen, Liang Zheng, Junxiong Ma

**Affiliations:** a Department of Orthopedics, General Hospital of Northern Theater Command of Chinese PLA, Shenyang, Liaoning Province, China.

**Keywords:** lumbar disc herniation, lumbar discectomy, posterior instrumented lumbar fusion, postoperative complications, postoperative discal pseudocyst

## Abstract

Postoperative discal pseudocyst (PDP) is rare. Few studies have reported on the recurrence of PDPs, and there is a lack of understanding of their clinical features and treatment methods. This study discusses the clinical characteristics and treatment of recurrent PDPs.

**Patient concerns:**

A 25-year-old man presented with radiating pain and numbness in the lateral left calf and dorsum of the foot.

**Diagnosis:**

Postoperative discal pseudocyst.

**Interventions:**

He underwent lumbar discectomy, which provided immediate postoperative relief. However, the symptoms recurred 45 days later. Magnetic resonance imaging (MRI) showed a lesion compressing the dura and nerve roots at the site of the previous surgery. The lesion appeared hypointense on T1-weighted imaging and hyperintense on T2-weighted imaging. The patient was treated conservatively for 1 month without significant relief. He then underwent lumbar discectomy and cyst removal, which immediately relieved his symptoms. However, 27 days later, the patient again developed the same symptoms. MRI examination showed recurrence of PDP. As 1 month of conservative treatment failed to relieve the patient’s symptoms, we performed posterior instrumented lumbar fusion and cyst removal.

**Outcomes:**

The patient’s symptoms disappeared, and have not recurred for 1 year at the time of writing.

**Conclusions:**

PDP is a rare complication of lumbar discectomy. Repeat lumbar discectomy can effectively treat PDP, but the cyst can recur. We, for the first time, used posterior instrumented lumbar fusion to successfully treat recurrent PDP.

## 1. Introduction

Some patients who undergo surgery for lumbar disc herniation develop recurrent radiating numbness and pain in the lower extremities after the surgery. On magnetic resonance imaging (MRI), these patients are found to have a cystic lesion compressing the dura and nerve roots in the spinal canal at the site of the surgery. The lesion exhibits low signal intensity on T1-weighted images and high signal intensity on T2-weighted images. This type of lesion is called a postoperative discal pseudocyst (PDP), and it was first reported by Young et al^[1]^ in 2009.

PDPs are rare. Kang and Park^[2]^ reported an incidence rate of approximately 1%, and found that most PDPs occurred following less-invasive lumbar discectomy procedures, such as percutaneous endoscopic discectomy, microdiscectomy, and microendoscopic discectomy. Few studies have reported on the recurrence of PDPs, and there is a lack of understanding of their clinical features and treatment methods. Here, we report a case of recurrent PDP in a patient who had previously undergone lumbar discectomy for lumbar disc herniation followed by surgical PDP removal. For treatment of the recurrent PDP, the patient again underwent surgical PDP removal along with posterior instrumented lumbar fusion, with good results. We review the related literature, and discuss the clinical characteristics of PDPs and the treatment of recurrent PDPs.

## 2. Case presentation

**Figure 1.**
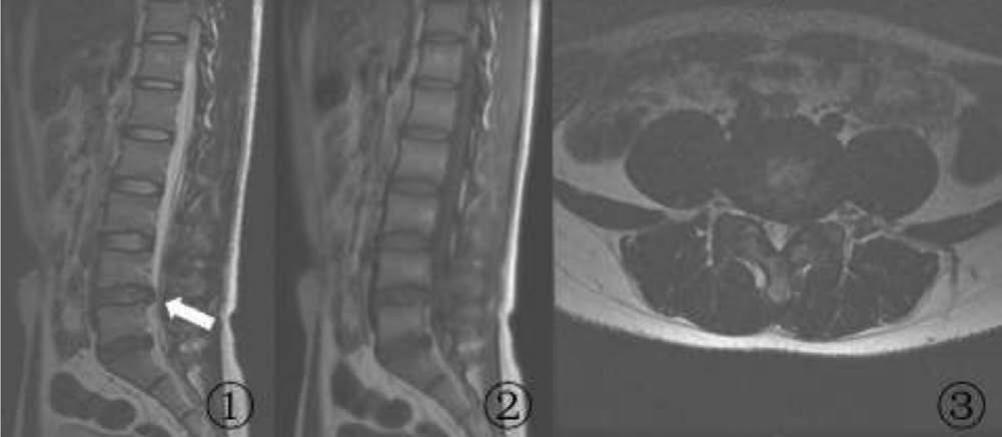
Preoperative lumbar MRI. A (A) sagittal T2-weighted image, (B) sagittal T1-weighted image, and (C) transverse T2-weighted image show the L4/5 disc protruding posteriorly and to the left, and compressing the left L5 nerve root. MRI = magnetic resonance imaging.

A 25-year-old man presented with a 1-year history of radiating pain and numbness in the left lower limb. The visual analogue scale (VAS) score was 6. The straight leg raise test produced pain when the patient actively lifted his left leg to 30°; the augmentation test was also positive. We detected hypesthesia of the skin over the left lateral calf, outer ankle, and dorsum of the foot. The strength of the left extensor hallucis longus was grade III; the remaining left lower-limb muscles had normal strength. The tendon reflexes of the left lower limb were normal. The right lower limb was normal. No lumbar instability was seen on lumbar frontal, lateral, and hyperextension-hyperflexion x-ray films. Computed tomography of the lumbar disc revealed that the L4/5 disc was herniated posteriorly and to the left without significant calcification. MRI of the lumbar spine confirmed that the L4/5 disc protruded posteriorly and to the left, and showed that the herniated disc compressed the left L5 nerve root (Fig. 1). A diagnosis of L4/5 disc herniation was established. The patient underwent L4/5 left lumbar discectomy under general anesthesia in the Orthopedic Department of the General Hospital of Northern Theater Command of Chinese PLA, and the symptoms of numbness and pain in the left lower limb were significantly relieved immediately after the surgery. The postoperative VAS score was 1.

**Figure 2.**
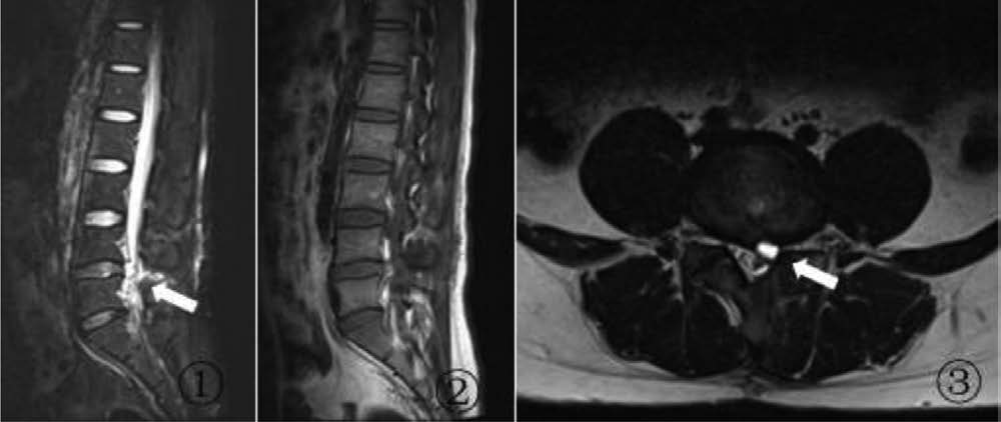
MRI of the lumbar spine after lumbar discectomy. A compressive cystic lesion with (A) high signal intensity on a sagittal T2-weighted image, (B) low signal intensity on a sagittal T1-weighted image, and (C) high signal intensity on a transverse T2-weighted image is seen at the site of the previous lumbar discectomy surgery. MRI = magnetic resonance imaging.

At 45 days after the surgery, the patient gradually developed radiating pain and numbness in the left lower limb along with painful claudication. The VAS score was 7. The active straight leg raising test was again positive at 30° on the left side, and the augmentation test was also positive. We detected hyperalgesia of the skin over the left lateral calf, external ankle, and dorsal foot. The strength of the left extensor hallucis longus was grade IV. MRI of the lumbar spine showed an epidural space-occupying lesion in the spinal canal at this site of the lumbar discectomy surgery. The lesion appeared hypointense on T1-weighted imaging and hyperintense (same intensity as cerebrospinal fluid) on T2-weighted imaging. The cyst compressed the dura and the left L5 nerve root, and communicated with the intravertebral disc (Fig. 2). Considering the above findings, we established a diagnosis of L4/5 PDP. Conservative treatment consisting of bed rest and pain relief was administered for 4 weeks, but the patient experienced no significant relief. Therefore, we performed L4/5 left lumbar discectomy and cyst removal. Intraoperatively, a cystic lesion containing old bloody fluid and measuring approximately 1.0 × 1.0 × 0.6 cm was observed in the posterior part of the disc at the site of the previous surgery. The pseudocyst was removed, and no disc protrusion or free nucleus pulposus tissue was seen. After the surgery, the patient experienced significant relief of the symptoms in the left lower extremity, and had a VAS score of 1.

**Figure 3.**
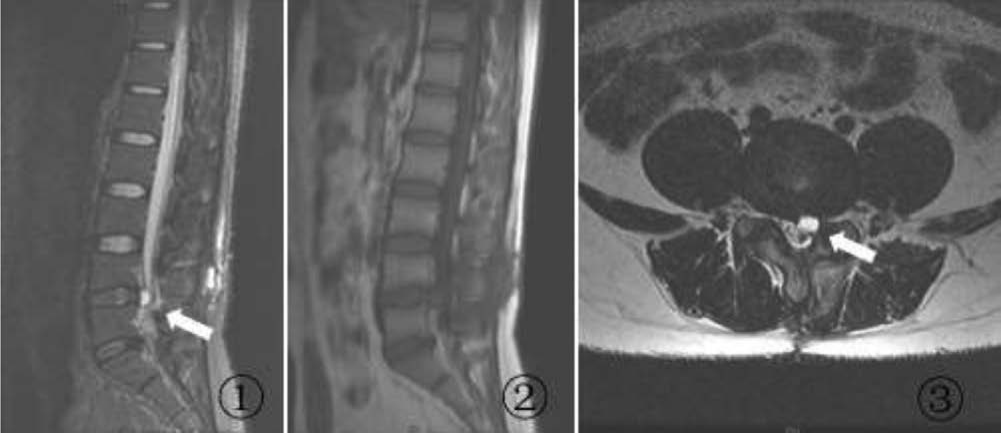
MRI of the lumbar spine after lumbar discectomy and cyst removal. A lesion that is larger than the previous one is seen in the original surgical area. The lesion shows (A) high signal intensity on a sagittal T2-weighted image, (B) low signal intensity on a sagittal T1-weighted image, and (C) high signal intensity on a transverse T2-weighted image. MRI = magnetic resonance imaging.

**Figure 4.**
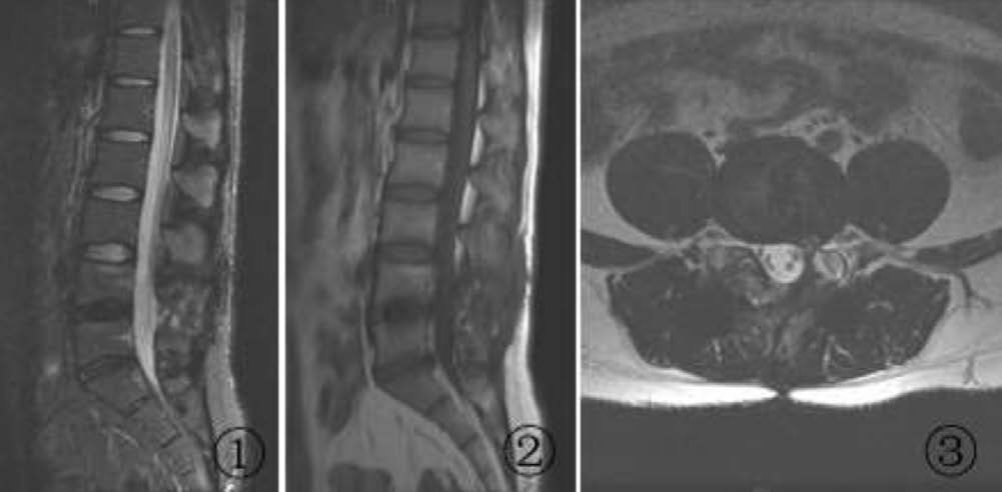
MRI after posterior instrumented lumbar fusion and cyst removal. A (A) sagittal T2-weighted image, (B) sagittal T1-weighted image, and (C) transverse T2-weighted image show the disappearance of the pseudocyst and no significant compression of the left L5 nerve root. MRI = magnetic resonance imaging.

At 27 days after the second surgery, the patient again presented with radiating pain and numbness in the left lower extremity. The VAS score was 7, and the findings of a physical examination were the same as the previous findings. MRI of the lumbar spine again showed a cystic lesion in the same location as before, but the lesion was enlarged compared with the previous cyst (Fig. 3). A diagnosis of recurrence of L4/5 PDP was established. Conservative treatment with bed rest, swelling reduction, and pain relief was administered for 26 days; however, the patient’s symptoms not only failed to improve but slightly worsened (VAS score, 8). Therefore, we decided to perform L4/5 posterior instrumented lumbar fusion and cyst removal for the patient. Intraoperatively, a cystic lesion measuring approximately 1.2 × 1.0 × 0.7 cm was seen in the original surgical area, with scarring and tissue adhesions obscuring the anatomical structures in the surgical field. The pseudocyst and the left articular eminence were removed, and L4-5 posterior instrumented lumbar fusion was performed. After the surgery, the patient’s left lower extremity symptoms were significantly relieved. The active straight leg raising test was negative, and the VAS score was 1. At a review examination 1 year after the last surgery, the patient had no discomfort in the left lower extremity, and MRI showed disappearance of the original pseudocyst and adequate nerve root decompression (Fig. 4).

## 3. Discussion and conclusions

### 3.1. Clinical features

Few studies have been conducted on PDP, and most available studies are case reports. We searched the PubMed database for the keywords “postoperative discal cyst,” “annular pseudocyst,” “discal pseudocyst,” and “post-discectomy pseudocyst.” We found 10 papers, including 1 paper from the United States^[1]^ and 9 papers from Asia.^[2–10]^ The findings of these 10 papers have been summarized in Table 1. These 10 studies and the present study included a total of 39 patients, of whom 94.87% (37 patients) were Asians, and 92.31% (36 patients) were men. Kang and Park^[2]^ reported that among 1503 patients who underwent surgery for lumbar disc herniation, 15 developed PDPs, yielding an incidence rate of approximately 1%. Chung et al^[3]^ reported 12 patients with PDPs; the mean age of the patients was 29 years, and 91.67% (11 patients) of the patients were male. The diagnosis of PDP can be made using MRI and discography. Discography is an invasive test that is rarely used nowadays. On MRI, PDPs^[2]^ appear hypointense on T1-weighted images and hyperintense on T2-weighted images, and are located at the site of the previous spinal surgery. This typical clinical presentation after lumbar disc surgery and characteristic MRI appearance are indicative of PDP, but the following should also be considered in the differential diagnosis: hematoma, discal cyst, lumbar facet joint synovial cysts, cyst of the ligamentum flavum, and synovial cyst of the spine.^[11–14]^ Most studies have reported that the onset of PDP occurs approximately 1–1.5 months after the surgery. Kang and Park^[2]^ and Chung et al^[3]^ recorded the time of onset of PDP based on the timing of the appearance of the MRI lesions; however, differences in the medical systems of the countries in which these studies were conducted may have had an impact on patient examination times and therefore affected the determination of the timing of PDP onset. In the present study, we judged the onset of PDP based on the appearance of lower limb symptoms after surgery. In this way, the effect of different examination times could be avoided. In the present study and in most of the published literature,^[1,2,4–10]^ PDPs have been documented to involve the L4/5 or L5/S1 level. Indeed, all 15 patients reviewed by Kang and Park^[2]^ had PDPs at 1 of these 2 levels, which appear to be the most commonly affected sites. The pathogenesis of PDP is unknown, but all patients presented after less-invasive surgical procedures such as lumbar discectomy and percutaneous endoscopic lumbar discectomy, which is consistent with our findings. Young et al^[1]^ suggested that the herniated disc fragments caused an inflammatory reaction around them, which led to the formation of a pseudocapsule. They considered that if the disc fragments were removed, but the pseudocapsule was not completely destroyed, fluid would collect inside the pseudocapsule and lead to the formation of a pseudocyst. Fu et al^[4]^ suggested that the surgical trauma of discectomy may accelerate the pathological progression of discal cysts.

### 3.2. Treatment methods

There is no consensus regarding the treatment of PDP. In 2009, Young et al^[1]^ reported the first 2 patients with PDPs; both were treated with percutaneous aspiration and steroid injection, which achieved good results within a short follow-up period. Chung et al^[3]^ reported 12 PDP patients, of whom 6 were treated conservatively, 1 was treated with percutaneous aspiration, and 5 were treated with surgery; all patients achieved good results. The time to cyst regression, as determined using review MRI, was highly variable (20–225 days) among the 6 patients who were treated conservatively. Furthermore, cyst regression and symptom improvement were closely correlated. In the study by Kang and Park,^[2]^ 10 of the 15 patients chose conservative treatment, while 5 patients underwent surgery. One of the conservatively treated patients was found to have an enlarged cyst on review MRI. All 15 patients showed some symptomatic improvement after treatment, but continued to have residual symptoms. The authors therefore concluded that conservative and surgical treatments did not differ from each other in terms of patient outcomes. Most authors^[2,3,5–7,9]^ believe that patients with PDPs can first be treated conservatively, and if conservative treatment is ineffective, surgery can be considered.

There are no reports in the literature on the treatment of PDP recurrence after resection. In our report, the patient underwent lumbar discectomy for the treatment of lumbar disc herniation and developed a PDP 1.5 months after the surgery. As 4 weeks of conservative treatment was ineffective, we performed lumbar discectomy again along with cyst removal. However, recurrence occurred about 4 weeks after the second surgery, possibly because the scarring from the first surgery obscured the surgical field and led to incomplete removal of the pseudocyst. Once again, conservative treatment was administered for 4 weeks, but was ineffective. Considering that the patient had undergone multiple surgeries, we presumed that the scarring and tissue adhesions in the operative area would be severe, and that further recurrences were likely if the cyst could not be completely removed. To avoid further recurrences and relieve the patient’s pain, we performed posterior instrumented lumbar fusion, which significantly alleviated the patient’s symptoms. A review MRI at 1 year after the last operation showed no recurrence. Therefore, we believe that patients with PDPs should first be offered conservative treatment. If this treatment is ineffective, or if the patient has decreased muscle strength in the lower extremities, surgical treatment such as lumbar discectomy is feasible. For recurrent PDP, posterior instrumented lumbar fusion is an effective treatment modality.

In summary, PDP occurs mainly in young Asian men, and has an incidence rate of approximately 1%. It commonly develops 1–1.5 months after lumbar disc surgery. PDP is mainly diagnosed using MRI, and is characterized by hypointensity on T1-weighted images and hyperintensity on T2-weighted images. The most commonly affected vertebral levels are the L4/5 and L5/S1 levels. The underlying etiology of PDP is currently unknown. These cysts are known to occur after less-invasive surgical procedures such as lumbar discectomy and percutaneous endoscopic lumbar discectomy. Most authors believe that PDPs should first be treated conservatively, and surgery is feasible if conservative treatment fails. For recurrent PDP, posterior instrumented lumbar fusion is an effective treatment modality.

**Table 1:** Summary of the published literature on PDPs.

Author	Patients (no.)	Sex	Country	Average age (yr)	Affected vertebral level	Previous operation	Time to MRI detection (d)	Treatment modality	Prognosis
Young et al^[1]^	2	M (2)	USA	49	L4/5 (1), L5/S1 (1)	MD (1), PD (1)	410	Percutaneous aspiration (1), steroid injection (1)	Symptoms relieved
Kang and Park^[2]^	15	M (15)	Korea	22.5	L4/5 (6), L5/S1 (9)	TFED (6), ILED (9)	53.7	Conservative (10), surgery (5)	Symptoms relieved
Chung et al^[3]^	12	M (11), F (1)	Korea	29.3	L2/3 (3), L4/5 (7), L5/S1 (2)	MD (9), PELD (3)	31.2	Conservative (6), surgery (5), needle aspiration (1)	Symptoms disappeared
Fu et al^[4]^	1	M	China	23	L4/5	PELD	40	Conservative	Symptoms disappeared
Prasad et al^[5]^	1	M	India	30	L4/5	NA	25	Surgery	Symptoms relieved
Jha et al^[6]^	2	M (1), F (1)	Japan	17	L4/5 (1), L5/S1 (1)	MED (2)	45	Conservative (2)	Symptoms relieved
Xu et al^[7]^	1	M	China	27	L5/S1	PEID	40	Surgery	Symptoms relieved
Li et al^[8]^	1	M	China	30	L4/5	PELD	37	Ozone ablation	Symptoms relieved
Manabe et al^[9]^	1	M	Japan	21	L4/5 (1)	PED	42	Surgery	Symptoms relieved
Shiboi et al^[10]^	2	M (1), F (1)	Japan	20.5	L4/5 (2)	PELD (2)	44.5	Surgery (2)	Symptoms relieved

F = female, ILED = interlaminar endoscopic discectomy, M = male, MD = microdiscectomy, MED = microendoscopic discectomy, NA = not available, PD = partial discectomy, PDP = postoperative discal pseudocyst, PED = percutaneous endoscopic discectomy, PEID = percutaneous endoscopic interlaminar discectomy, PELD = percutaneous endoscopic lumbar discectomy, TFED = transforaminal endoscopic discectomy.

## Acknowledgements

We thank Medjaden Inc. for scientific editing of this manuscript.

## Author Contributions

**Conceptualization:** Hong Wang, Junxiong Ma.

**Data curation:** Hong Wang, Shuang Wang, Liang Zheng, Hailong Yu, Yu Chen, Junxiong Ma.

**Formal analysis:** Hong Wang.

**Investigation:** Hong Wang, Shuang Wang, Liang Zheng, Hailong Yu, Yu Chen, Junxiong Ma.

**Methodology:** Hong Wang, Junxiong Ma.

**Supervision:** Hong Wang, Junxiong Ma.

**Validation:** Hong Wang, Junxiong Ma.

**Visualization:** Hong Wang, Shuang Wang, Liang Zheng, Hailong Yu, Yu Chen, Junxiong Ma.

**Writing—original draft:** Hong Wang, Shuang Wang, Liang Zheng, Hailong Yu, Yu Chen, Junxiong Ma.

**Writing—review & editing:** Hong Wang, Shuang Wang, Liang Zheng, Hailong Yu, Yu Chen, Junxiong Ma.

## References

[1] Young PM Fenton DS Czervionke LF. Postoperative annular pseudocyst: report of two cases with an unusual complication after microdiscectomy, and successful treatment by percutaneous aspiration and steroid injection. Spine J. 2009;9:e9–e15. 10.1016/j.spinee.2007.12.013 18280218

[2] Kang SH Park SW. Symptomatic post-discectomy pseudocyst after endoscopic lumbar discectomy. J Korean Neurosurg Soc. 2011;49:31–6. 21494360 10.3340/jkns.2011.49.1.31 PMC3070892

[3] Chung D Cho DC Sung JK. Retrospective report of symptomatic postoperative discal pseudocyst after lumbar discectomy. Acta Neurochir (Wien). 2012;154:715–22. 22223287 10.1007/s00701-011-1219-7

[4] Fu CF Tian ZS Yao LY. Postoperative discal pseudocyst and its similarities to discal cyst: a case report. World J Clin Cases. 2021;9:1439–45. 33644213 10.12998/wjcc.v9.i6.1439 PMC7896671

[5] Prasad GL Menon GR. Post-discectomy annular pseudocyst: a rare cause of failed back syndrome. Neurol India. 2017;65:650–2. 28488645 10.4103/neuroindia.NI_558_16

[6] Jha SC Tonogai I Higashino K. Postoperative discal cyst: an unusual complication after microendoscopic discectomy in teenagers. Asian J Endosc Surg. 2016;9:89–92. 26781537 10.1111/ases.12227

[7] Xu WB Wu DJ Chen C. Symptomatic postoperative discal pseudocyst after percutaneous endoscopic interlaminar discectomy: case report and literature review. Orthop Surg. 2021;13:347–52. 33331078 10.1111/os.12863 PMC7862141

[8] Li J Liang S Xie W. Symptomatic postoperative discal pseudocyst following percutaneous endoscopic lumbar discectomy: a case report and review of the literature. Medicine (Baltim). 2021;100:e24026. 10.1097/MD.0000000000024026 PMC7837847 33545999

[9] Manabe H Higashino K Sugiura K. A rare case of a discal cyst following percutaneous endoscopic lumbar discectomy via a transforaminal approach. Int J Spine Surg. 2019;13:92–4. 30805291 10.14444/6012 PMC6383457

[10] Shiboi R Oshima Y Kaneko T. Different operative findings of cases predicted to be symptomatic discal pseudocysts after percutaneous endoscopic lumbar discectomy. J Spine Surg. 2017;3:233–7. 28744506 10.21037/jss.2017.05.07 PMC5506303

[11] Chiba K Toyama Y Matsumoto M. Intraspinal cyst communicating with the intervertebral disc in the lumbar spine: discal cyst. Spine (Phila Pa 1976). 2001;26:2112–8. 11698889 10.1097/00007632-200110010-00014

[12] Apostolaki E Davies AM Evans N. MR imaging of lumbar facet joint synovial cysts. Eur Radiol. 2000;10:615–23. 10795544 10.1007/s003300050973

[13] Asamoto S Jimbo H Fukui Y. Cyst of the ligamentum flavum. Neurol Med Chir (Tokyo). 2005;45:653–6. 16377956 10.2176/nmc.45.653

[14] Onofrio BM Mih AD. Synovial cysts of the spine. Neurosurgery. 1988. 10.1227/00006123-198804000-00004 3374775

